# Supplementary material for: Complex‐centric proteome profiling by SEC‐SWATH‐MS
Source: Mol Syst Biol. 2019 Jan 14;15(1):e8438. doi: 10.15252/msb.20188438 (PMC6346213; doi:10.15252/msb.20188438)
Supplement: Supplementary file 8 — Dataset EV7 [file MSB-15-e8438-s008.zip › feature_plots_string/Q8TEM1.pdf]

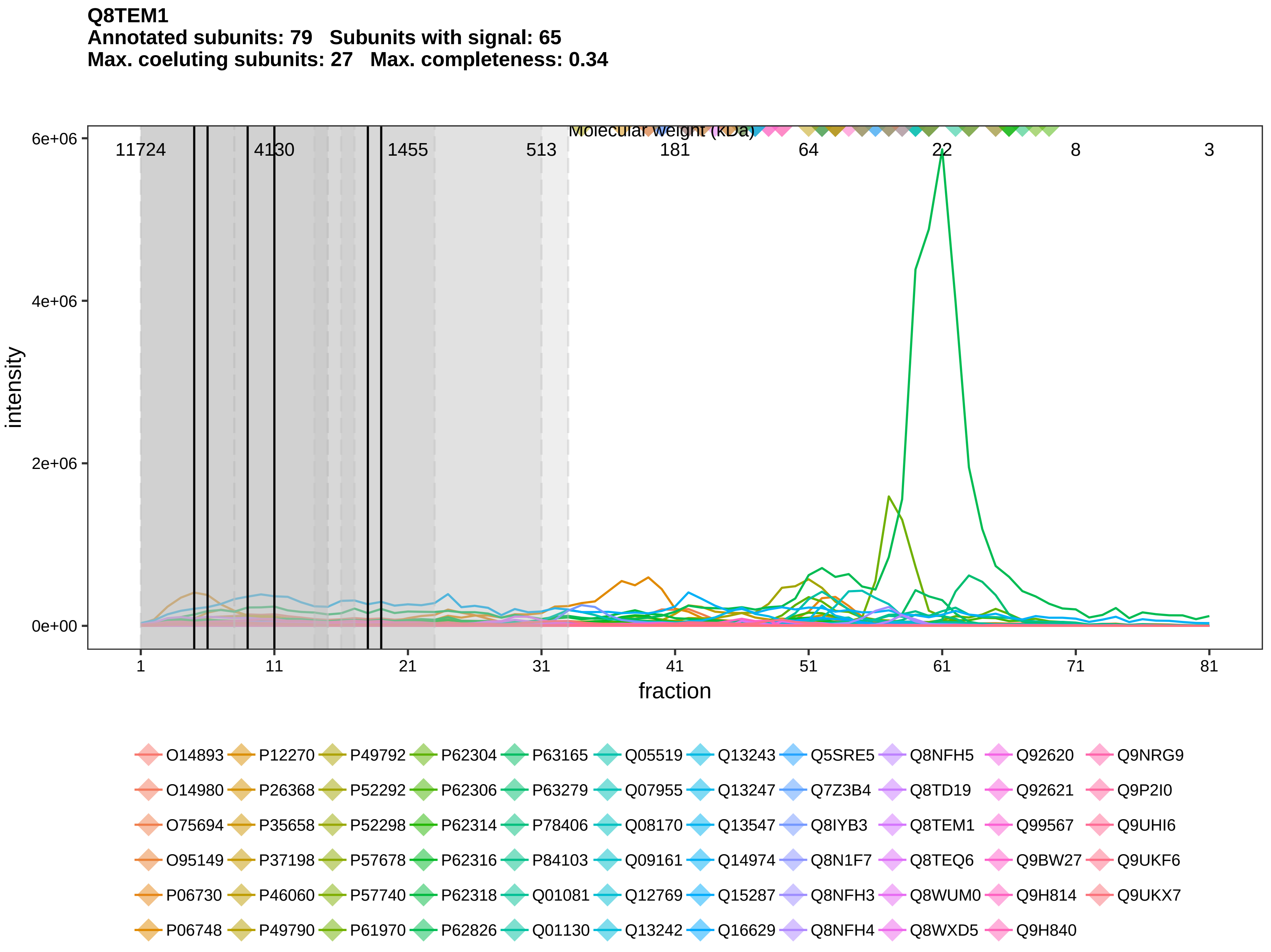
Q8TEM1
Annotated subunits: 79 Subunits with signal: 65
Max. coeluting subunits: 27 Max. completeness: 0.34
Molecular weight (kDa)
11724
4130
1455
513
181
64
22
8
3
intensity
6e+06
4e+06
2e+06
0e+00
fraction
1
11
21
31
41
51
61
71
81
O14893 P12270 P49792 P62304 P63165 Q05519 Q13243 Q5SRE5 Q8NFH5 Q92620 Q9NRG9
O14980 P26368 P52292 P62306 P63279 Q07955 Q13247 Q7Z3B4 Q8TD19 Q92621 Q9P2I0
O75694 P35658 P52298 P62314 P78406 Q08170 Q13547 Q8IYB3 Q8TEM1 Q99567 Q9UHI6
O95149 P37198 P57678 P62316 P84103 Q09161 Q14974 Q8N1F7 Q8TEQ6 Q9BW27 Q9UKF6
P06730 P46060 P57740 P62318 Q01081 Q12769 Q15287 Q8NFH3 Q8WUM0 Q9H814 Q9UKX7
P06748 P49790 P61970 P62826 Q01130 Q13242 Q16629 Q8NFH4 Q8WXD5 Q9H840
